# Relaxation Exponents of OTOCs and Overlap with Local Hamiltonians

**DOI:** 10.3390/e25010059

**Published:** 2022-12-28

**Authors:** Vinitha Balachandran, Dario Poletti

**Affiliations:** 1 Science, Mathematics and Technology Cluster, Singapore University of Technology and Design, 8 Somapah Road, Singapore 487372, Singapore; 2 EPD Pillar, Singapore University of Technology and Design, 8 Somapah Road, Singapore 487372, Singapore; 3 MajuLab, CNRS-UCA-SU-NUS-NTU International Joint Research Unit, Singapore 117543, Singapore; 4 Centre for Quantum Technologies, National University of Singapore, Singapore 117543, Singapore; 5 The Abdus Salam International Centre for Theoretical Physics, Strada Costiera 11, 34151 Trieste, Italy

**Keywords:** OTOC, information scrambling, relaxation dynamics

## Abstract

OTOC has been used to characterize the information scrambling in quantum systems. Recent studies have shown that local conserved quantities play a crucial role in governing the relaxation dynamics of OTOC in non-integrable systems. In particular, the slow scrambling of OTOC is seen for observables that have an overlap with local conserved quantities. However, an observable may not overlap with the Hamiltonian but instead with the Hamiltonian elevated to an exponent larger than one. Here, we show that higher exponents correspond to faster relaxation, although still algebraic, and such exponents can increase indefinitely. Our analytical results are supported by numerical experiments.

## 1. Introduction

For generic many-body quantum systems, information initially encoded in a few local degrees of freedom can spread in time over the entire accessible space. This process is called information scrambling and can be characterized by out-of-time ordered correlators (OTOCs) [1,2,3,4,5,6,7,8,9,10,11,12,13,14,15,16,17,18,19,20,21]. For quantum systems with a classical limit, OTOCs can be mapped to Lyapunov exponents [22,23,24,25,26,27,28,29,30,31,32,33]. Because of this, OTOCs have been applied to understand the thermalization in many-body quantum systems [3,4,5,6,7,8,9,10,11,12,13].

Recent studies have pointed out the relevance of local conserved quantities in the relaxation dynamics of OTOCs [34,35,36,37,38,39,40]. In particular, in [39] it was shown that the emergence of algebraic relaxation can stem from the locality of the Hamiltonian, i.e., the ensuing presence of a Lieb–Robinson bound [41], and the eigestate thermalization hypothesis (ETH) [42,43]. Importantly, with the approach developed in [39] it was also possible to show that the algebraic relaxation of the OTOC is typical.

In the scenarios considered until now, the operators in the OTOC had non-zero overlap with the Hamiltonian or a local conserved quantity (i.e., total magnetization). Here, we investigate how the relaxation dynamics would be affected if the operators in the OTOC, e.g., *A*, do not overlap with the Hamiltonian *H* but only with one of its powers, i.e., tr(AH)=0, but $\mathrm{tr}(AH^{m})\neq 0$ for *m*, being an integer larger than one. We show that depending on the exponent *m* at which the overlap becomes non-zero, we expect an algebraic relaxation of the OTOC in time with an exponent proportional to *m*. To obtain this result, we also show the relation between the first non-zero derivative of the diagonals of an operator in the energy basis, with the exponent *m* at which $\mathrm{tr}(AH^{m})\neq 0$.

The paper is organized as follows. In Section 2, we introduce the definition of OTOCs and explain the relaxation dynamics of OTOCs from the knowledge of the matrix elements of the observables in the eigenenergy basis. In Section 3, we show analytically our main result, i.e., that any different exponents can emerge in the relaxation of the OTOC, depending on the order at which the operators in the OTOC overlap with the Hamiltonian. Our numerical results are presented in Section 4. We draw our conclusions in Section 5.

## 2. Emergence of Slow Scrambling

### 2.1. Definition

Consider the infinite-temperature out-of-time-ordered correlator (OTOC) between two local observables *A* and *B* defined as

(1) $$\begin{array}{cc} O^{AB}\left(t\right) & =\frac{1}{2}\langle \left[A\left(t\right),B\right]{\left[A\left(t\right),B\right]}^{†}\rangle \end{array}$$

where $A\left(t\right)=U^{†}AU$ is the time evolved operator *A* due to the unitary evolution $U=Te^{-i\int_{0}^{t}H\left(\tau \right)d\tau }$ from the time-ordered integration of the (generically) time-dependent Hamiltonian H(t). Expanding the commutators, we can rewrite Equation (1) as

(2) $$\begin{array}{cc} \frac{1}{2}\langle \left[A\left(t\right),B\right]{\left[A\left(t\right),B\right]}^{†}\rangle & =\langle B^{2}A{\left(t\right)}^{2}\rangle -\langle A\left(t\right)BA\left(t\right)B\rangle \\ \; & =G^{AB}\left(t\right)-F^{AB}\left(t\right), \end{array}$$

where $G^{AB}\left(t\right)=\langle B^{2}A{\left(t\right)}^{2}\rangle$ is the time-ordered part of OTOC and $F^{AB}\left(t\right)=\langle A\left(t\right)BA\left(t\right)B\rangle$ is the not-time-ordered part. We consider only unitary and Hermitian observables for which G(t)=1, and hence we restrict ourselves to F(t) in the remaining part. Taking energy eigenstates as the basis of the Hilbert space, the time evolution of OTOC can be written in the eigenenergy basis |p〉 as

(3) $$\begin{array}{c} F^{AB}\left(t\right)=\frac{1}{V}\sum_{p,q,k,l}e^{i(E_{p}-E_{q}+E_{k}-E_{l})t}A_{pq}B_{qk}A_{kl}B_{lp} \end{array}$$

where $E_{p}$ is the eigenenergy, $A_{pq}=\langle p|A|q\rangle$, and $B_{qk}=\langle q|B|k\rangle$. We work in units for which ℏ=1.

As t→∞, dominant terms in the above expression are those for which $E_{p}-E_{q}+E_{k}-E_{l}=0$. Hence, for generic systems [44,45], the infinite-time value of $F^{AB}\left(t\right)$ is given by

(4) $$\begin{array}{cc} F^{AB}\left(\infty \right)=\frac{1}{V}(\sum_{p} & A_{pp}^{2}B_{pp}^{2}+\sum_{p,q\neq p}(A_{pp}B_{pq}A_{qq}B_{qp}\\ + & A_{pq}B_{qq}A_{qp}B_{pp})). \end{array}$$

Equation (4) highlights the importance of diagonal elements of *A* and *B* in the eigenenergy basis in the infinite-time value of OTOC. Indeed, a non-zero diagonal element in *A* or *B* is necessary to guarantee a non-zero value of $F^{AB}\left(\infty \right)$.

### 2.2. Conditions for Algebraic Relaxation of OTOC

Two sufficient conditions for the emergence of algebraic relaxation of OTOC [39,40] are

- A Lieb–Robinson bound (or even an algebraic spreading of correlation that occurs in systems with power-law interactions),
- The algebraic scaling of the infinite-time value of the OTOC with the system size.

In local and bounded Hamiltonians, the speed of propagation of the correlations is limited by Lieb–Robinson bound [41,46]. Hence, an accurate description of the evolution of OTOC of a thermodynamically large system can be obtained simply considering a finite portion of it. Assuming that the system is maximally scrambled within the region of size *L*, the decay of $F_{L=\infty }^{AB}\left(t\right)$ is bounded by the Lieb–Robinson velocity $v_{\mathrm{LR}}$ as

(5) $$\begin{array}{c} F_{L=\infty }^{AB}\left(t\right)\approx F_{L=s\;v_{\mathrm{LR}}\;t}^{AB}\left(\infty \right), \end{array}$$

where *s* is a real number larger than 1. Hence, *L* increases with time and is a time-dependent quantity. Therefore, the scaling of $F_{L}^{AB}\left(\infty \right)$ is crucial to predict the bound for the relaxation of OTOC. In particular, when $F_{L}^{AB}\left(\infty \right)$ decays algebraically with the system size, e.g., $F_{L}^{AB}\left(\infty \right)\propto L^{-\alpha }$, then the OTOC of the thermodynamic size system cannot decay faster than algebraically in time, or more precisely from Equation (5) one can write that it cannot be faster than

(6) $$\begin{array}{c} F_{L=\infty }^{AB}\left(t\right)\propto \frac{1}{t^{\alpha }} \end{array}$$

because $L=s\;v_{\mathrm{LR}}\;t$.

The actual decay of the OTOC may even be slower, for example, considering cases in which the system goes through prethermalization [47] or in which the system is many-body localized [48]. However, the relaxation cannot be faster; hence, the OTOC will have a slow, non-exponential relaxation. A comprehensive analysis of this is presented in [39].

## 3. Generic Algebraic Relaxation in Short-Ranged Systems

### 3.1. Estimate of the Infinite Time Value of OTOC

In this section, we show how to obtain the approximate value of the infinite-time, finite-size, OTOC $F_{L}^{AB}\left(\infty \right)$

(7) $$\begin{array}{cc} F_{L}^{AB}\left(\infty \right)= & \frac{1}{V}\sum_{p}A_{pp}^{2}B_{pp}^{2}+\frac{1}{V}\sum_{p,q\neq p}A_{pp}A_{qq}{\left|B_{pq}\right|}^{2}\\ \; & +\frac{1}{V}\sum_{p,q\neq p}B_{pp}B_{qq}{\left|A_{pq}\right|}^{2}\\ \approx & \frac{1}{V}\sum_{p}A_{pp}^{2}B_{pp}^{2}+\frac{1}{V}\sum_{p}A_{pp}A_{pp}\left[{\left(BB^{†}\right)}_{pp}-B_{pp}^{2}\right]\\ \; & +\frac{1}{V}\sum_{p}B_{pp}B_{pp}\left[{\left(AA^{†}\right)}_{pp}-A_{pp}^{2}\right]\\ \approx & \frac{1}{V}\sum_{p}A_{pp}^{2}B_{pp}^{2}+\frac{1}{V}\sum_{p}\left[\mathrm{tr}\left(BB^{†}\right)-B_{pp}^{2}\right]A_{pp}^{2}\\ \; & +\frac{1}{V}\sum_{p}\left[\mathrm{tr}\left(AA^{†}\right)-A_{pp}^{2}\right]B_{pp}^{2}\\ \approx & \frac{1}{V}\sum_{p}A_{pp}^{2}B_{pp}^{2}+\frac{1}{V}\sum_{p}\left[1-B_{pp}^{2}\right]A_{pp}^{2}\\ \; & +\frac{1}{V}\sum_{p}\left[1-A_{pp}^{2}\right]B_{pp}^{2}\\ \approx & \frac{1}{V}\sum_{p}\left[A_{pp}^{2}+B_{pp}^{2}-A_{pp}^{2}B_{pp}^{2}\right]\\ \approx & \frac{1}{V}\sum_{p}\left[A_{pp}^{2}+B_{pp}^{2}\right], \end{array}$$

where we have used steps similar to [39,45], and a similar discussion can be found in [40]. Thus, the main contribution of the infinite-time finite-size OTOC comes from the $A_{pp}^{2}$ and $B_{pp}^{2}$ terms, which we will be discussing in the following.

### 3.2. Structure of the Diagonal Elements

In short, the diagonal element $A_{pp}$ can be approximated by a function of eigenenergy $E_{p}$

(8) $$\begin{array}{cc} |A_{pp}-f_{A}\left(E_{p}/L\right)| & \leq e^{-(\Omega (L))}, \end{array}$$

where $f_{A}\left(E_{p}/L\right)$ can be expanded as

(9) $$\begin{array}{cc} f_{A}\left(E_{p}/L\right) & =f_{A}\left(0\right)+f_{A}^{\left(1\right)}\left(0\right)E_{p}/L+\frac{1}{2}f_{A}^{\left(2\right)}\left(0\right)E_{p}^{2}/L^{2}+...\\ \; & =\sum_{q}\frac{f_{A}^{\left(q\right)}}{q!}{\left(\frac{E_{p}}{L}\right)}^{q} \end{array}$$

with $f_{A}^{\left(q\right)}$ being the *q*-th derivative of $f_{A}$. We also note that, using Lemma 1 in [45], one can write

(10) $$\begin{array}{cc} \frac{1}{V}\sum_{p}E_{p}^{q} & =\langle H^{q}\rangle =O\left(L^{q/2}\right). \end{array}$$

In [45] it was shown that, for traceless operators $f_{A}\left(0\right)=0$, and if $f_{A}^{\left(1\right)}\left(0\right)\neq 0$, then we can write

(11) $$\begin{array}{cc} \mathrm{tr}\left(AH\right) & =\frac{1}{V}\sum_{p}A_{pp}E_{p}\\ \; & \approx \frac{1}{VL}\sum_{p}E_{p}^{2}f_{A}^{\left(1\right)}\left(0\right)\\ \; & \approx \frac{\langle H^{2}\rangle }{L}f_{A}^{\left(1\right)}\left(0\right) \end{array}$$

and thus

(12) $$\begin{array}{cc} f_{A}^{\left(1\right)}\left(0\right) & \approx \frac{\mathrm{tr}\left(AH\right)L}{\langle H^{2}\rangle }. \end{array}$$

Hence, the first derivative of a local observable *A* is independent of the system size. From Equation (12), we obtain

(13) $$\begin{array}{cc} F_{L}^{AB}\left(\infty \right) & \approx \frac{1}{V}\sum_{p}\left(A_{pp}^{2}+B_{pp}^{2}\right)\\ \; & \approx \frac{1}{V}\sum_{p}\frac{E_{p}^{2}}{L^{2}}\left[{\left(f_{A}^{\left(1\right)}\left(0\right)\right)}^{2}+{\left(f_{B}^{\left(1\right)}\left(0\right)\right)}^{2}\right]\\ \; & \approx \frac{1}{V}\sum_{p}\frac{E_{p}^{2}}{L^{2}}\frac{\left[\mathrm{tr}{\left(AH\right)}^{2}+\mathrm{tr}{\left(BH\right)}^{2}\right]L^{2}}{{\langle H^{2}\rangle }^{2}}\\ \; & \approx \frac{\mathrm{tr}{\left(AH\right)}^{2}+\mathrm{tr}{\left(BH\right)}^{2}}{\langle H^{2}\rangle }\\ \; & \propto \frac{1}{L}. \end{array}$$

The last step stems from the fact that tr(AH) and tr(BH) are independent of the system size, while $\langle H^{2}\rangle \propto L$ from Equation (10).

If tr(AH)=0 but, for instance, $\mathrm{tr}(AH^{p})\neq 0$ only for $p\geq p_{c}$ then one can generalize the previous result. Considering $f_{A}^{\left(q\right)}\left(0\right)$ as the smalles non-zero derivative of $f_{A}$ at zero energy (with the same parity as $p_{c}$), then we can write

(14) $$\begin{array}{cc} \mathrm{tr}(AH^{p_{c}}) & =\sum_{n}\frac{f_{A}^{\left(q\right)}}{q!}\frac{E_{n}^{q+p_{c}}}{L^{q}} \end{array}$$

which implies that

(15) $$\begin{array}{cc} f_{A}^{\left(q\right)} & =\frac{q!\mathrm{tr}\left(AH^{p_{c}}\right)L^{q}}{\langle H^{p_{c}+q}\rangle }. \end{array}$$

Now, if $q<p_{c}$ then $f_{A}^{\left(q\right)}$ would decay as $L^{-(p_{c}-q)/2}$, which implies that they are 0, and the non-size dependent $f_{A}^{\left(q\right)}\left(0\right)$ would occur exactly at $q=p_{c}$. This implies that the first non-zero derivative of $f_{A}\left(0\right)$ is the $p_{c}$-th one. Thus, when $\mathrm{tr}(AH^{p})\neq 0$ only for $p\geq p_{c}$ we can write

(16) $$\begin{array}{cc} F_{L}^{AB}\left(\infty \right) & \approx \frac{1}{V}\sum_{n}{\left(\frac{E_{n}}{L}\right)}^{2p_{c}}\left[{\left(f_{A}^{\left(p_{c}\right)}\left(0\right)\right)}^{2}+{\left(f_{B}^{\left(p_{c}\right)}\left(0\right)\right)}^{2}\right]\\ \; & \approx \frac{1}{V}\sum_{n}{\left(\frac{E_{n}}{L}\right)}^{2p_{c}}\frac{\left[\mathrm{tr}{\left(AH^{p_{c}}\right)}^{2}+\mathrm{tr}{\left(BH^{p_{c}}\right)}^{2}\right]L^{2p_{c}}}{{\langle H^{2p_{c}}\rangle }^{2}}\\ \; & \approx \frac{\mathrm{tr}{\left(AH^{p_{c}}\right)}^{2}+\mathrm{tr}{\left(BH^{p_{c}}\right)}^{2}}{\langle H^{2p_{c}}\rangle }\\ \; & \propto \frac{1}{L^{p_{c}}}. \end{array}$$

Building on Equation (16), and combining it with the Lieb–Robinson bound $L=s\;v_{\mathrm{LR}}\;t$, we can thus guarantee that $F^{AB}$ cannot relax faster than $t^{-p_{c}}$. Furthermore, for systems in which correlations mostly spread diffusively, i.e., proportional to $t^{1/2}$, we can can expect $F^{AB}$ to relax as $t^{-p_{c}/2}$. Hence, the structure of the diagonal elements of the observables, which is the first non-zero derivative at 0 energy, i.e., which is the first exponent of the Hamiltonian that has non-zero overlap with the operators *A* and *B* considered, plays an important role in the relaxation dynamics of the OTOC in the system. This is numerically verified in detail in the following section.

## 4. Results

### 4.1. Model

We consider a prototypical non-integrable model, the tilted Ising chain with Hamiltonian

(17) $$\begin{array}{cc} H & =\sum_{l=1}^{L-1}J_{z}\sigma_{l}^{z}\sigma_{l+1}^{z}+\sum_{l=1}^{L}\left(h_{x}\sigma_{l}^{x}+h_{z}\sigma_{l}^{z}\right), \end{array}$$

where $J_{z}$ is the coupling constant in the z direction, while $h_{x}$ and $h_{z}$ are the transverse and the longitudinal field strengths. The model is integrable when either $h_{x}=0$ or $h_{z}=0$. This can be verified by studying the level spacing statistics, which typically follows a Poisson distribution for integrable systems and a Wigner–Dyson distribution for non-integrable ones [49,50]. In particular, $\delta_{n}=E_{n+1}-E_{n}$, the level spacing between two consecutive energy levels $E_{n}$ and $E_{n+1}$ within a single symmetry sector, define the ratio $r_{n}=\max\left(\delta_{n},\delta_{n+1}\right)/\min\left(\delta_{n},\delta_{n+1}\right)$ and take an average $r=\sum_{n}r_{n}/N$, where *N* is the number of energy level differences considered. For a Poisson distribution, *r* can be computed analytically, and it gives r=2ln2−1≈0.386, while for a Wigner–Dyson distribution *r* can be evaluated numerically to be r≈0.529 [51]. In the current work, we use parameters $J_{z}=1$, $h_{z}=0.809$, and $h_{x}=0.9$, which result in r≈0.53 already for a system size of L=12 spins.

### 4.2. Observables and Structure of Their Diagonal Elements

**Figure 1:**
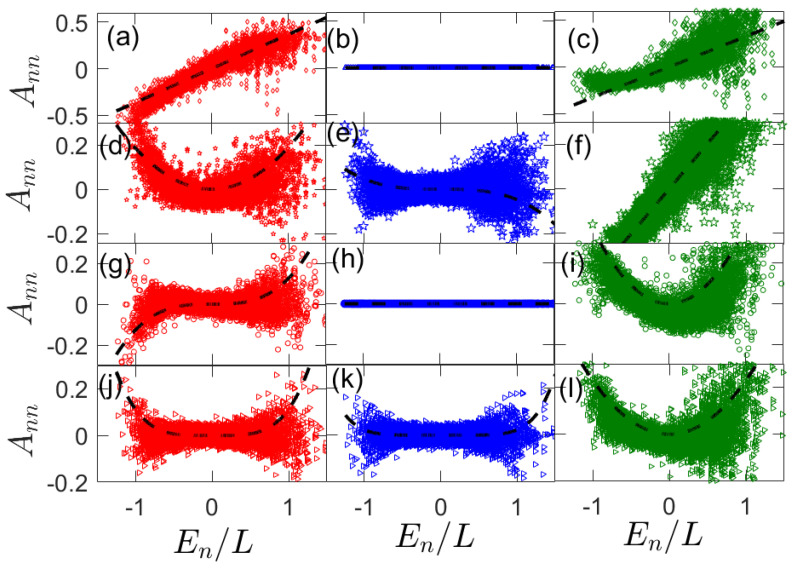
Diagonal elements of the observable in the energyeigen basis for single-site observables $A=\sigma_{L/2}^{\alpha }$ panel (**a**–**c**), double-site observables $A=\sigma_{L/2}^{\alpha }\sigma_{L/2+1}^{\alpha }$ (**d**–**f**), triple-site observables $A=\sigma_{L/2-1}^{\alpha }\sigma_{L/2}^{\alpha }\sigma_{L/2+1}^{\alpha }$ (**g**–**i**), and quadruple-site observables $A=\sigma_{L/2-2}^{\alpha }\sigma_{L/2-1}^{\alpha }\sigma_{L/2}^{\alpha }\sigma_{L/2+1}^{\alpha }$ (**j**–**l**). Left panels are for $\sigma_{l}^{x}$ (α=x) observables, middle panels are for $\sigma_{l}^{y}$ (α=y) observables, and right panels are for $\sigma_{l}^{z}$ (α=z) observables. Dashed lines are the lowest order fits in the Taylor expansion of the observable in Equation (9). Here, L=14, $J_{z}=1$, $h_{x}=0.9$, and $h_{z}=0.809$.

To span over a variety of different structures, and to have operators *A*, which have $\mathrm{tr}(AH^{p})\neq 0$ only for $p\leq p_{c}$ with $p_{c}$, which can be different from 1, we analyze both single-site and multi-site observables in our study. In particular, we consider the following four types of observables:

(18) $$\begin{array}{cc} \mathrm{single}-\mathrm{site}\to & \;\;\sigma_{l}^{\alpha } \end{array}$$

(19) $$\begin{array}{cc} \mathrm{double}-\mathrm{site}\to & \;\;\sigma_{l}^{\alpha }\sigma_{l+1}^{\alpha } \end{array}$$

(20) $$\begin{array}{cc} \mathrm{triple}-\mathrm{site}\to & \;\;\sigma_{l-1}^{\alpha }\sigma_{l}^{\alpha }\sigma_{l+1}^{\alpha } \end{array}$$

(21) $$\begin{array}{cc} \mathrm{quadruple}-\mathrm{site}\to & \;\;\sigma_{l-2}^{\alpha }\sigma_{l-1}^{\alpha }\sigma_{l}^{\alpha }\sigma_{l+1}^{\alpha } \end{array}$$

where α=x,y or *z*. The diagonal elements of these operators in the eigenbasis of Hamiltonian Equation (17) are shown in Figure 1. The left column is for α=x, the center column is for α=y, and the right column is for α=z. The rows are for increasing the range of operators from top to bottom, with the top row for single-site operators and the fourth row for four-site operators. In all of the panels, the dashed lines represent the expected algebraic energy dependence of $f_{A}$ near energy zero from Section 3.2. We note that these fits are evaluated directly from calculating $f_{A}^{\left(n\right)}\left(0\right)$ with Equation (15) along with eigenenergies $E_{n}$ for the system Hamiltonian in Equation (17) with no fitting parameters.

For single-site observables $A=\sigma_{l}^{x}$ and $\sigma_{l}^{z}$, and for the non-integrable Ising chain tr(AH)≠0 and so $f_{A}^{\left(1\right)}\left(0\right)\neq 0$. However, with $A=\sigma_{l}^{y}$, $\mathrm{tr}(AH^{n})=0$ for any *n*. Hence, we expect a linear variation of the diagonal elements of $\sigma_{l}^{x}$ and $\sigma_{l}^{z}$ with energy density $E_{n}/L$ and a flat profile for $\sigma_{l}^{y}$. This can be seen in Figure 1a–c. To conform our analytical predictions, we plot $f_{A}^{\left(1\right)}\left(0\right)E_{n}/L$ where $f_{A}^{\left(1\right)}\left(0\right)$ is calculated explicity form Equation (12).

The two-site observables $A=\sigma_{L/2}^{\alpha }\sigma_{L/2+1}^{\alpha }$, are shown in the panels (d–f). For $A=\sigma_{L/2}^{x}\sigma_{L/2+1}^{x}$, $\mathrm{tr}(AH^{2})\neq 0$, whereas tr(AH)=0 and, as predicted in Section 3.2, we thus observe that $f_{A}$ can be fitted by a parabola $f_{A}^{\left(2\right)}\left(0\right)E_{n}^{2}/\left(2!L^{2}\right)$ indicated by the dashed black lines. Since tr(AH)≠0 for $A=\sigma_{L/2}^{z}\sigma_{L/2+1}^{z}$, we see a linear scaling of $A_{nn}$ with $E_{n}/L$. For the $A=\sigma_{L/2}^{y}\sigma_{L/2+1}^{y}$ observable, $\mathrm{tr}(AH^{m})\neq 0$ for m≥3. Hence, we see a cubic structure of the diagonal elements with a fitting of the form $f_{A}^{\left(3\right)}\left(0\right)E_{n}^{3}/\left(3!L^{3}\right)$.

We also consider triple-site observables $A=\sigma_{L/2-1}^{\alpha }\sigma_{L/2}^{\alpha }\sigma_{L/2+1}^{\alpha }$. These are depicted in the panels (g–i). Here, $\mathrm{tr}(AH^{m})\neq 0$ for m≥3 for $\sigma_{l}^{x}$ observables, and we clearly see a cubic structure for the diagonal elements that can be fitted with lines of the form $f_{A}^{\left(3\right)}\left(0\right)E_{n}^{3}/\left(3!L^{3}\right)$. Since there are no diagonal elements for any power of *H* for the $\sigma^{y}$ observable, a flat profile is seen. With the $\sigma^{z}$ observable, a parabolic structure is seen since $\mathrm{tr}(AH^{2})\neq 0$, whereas tr(AH)=0. This is also nicely fitted by $f_{A}^{\left(2\right)}\left(0\right)E_{n}^{2}/\left(2!L^{2}\right)$ in panel (i).

For the four site observable, we study $A=\sigma_{L/2-2}^{\alpha }\sigma_{L/2-1}^{\alpha }\sigma_{L/2}^{\alpha }\sigma_{L/2+1}^{\alpha }$. For $A=\sigma_{L/2-2}^{x}\sigma_{L/2-1}^{x}\sigma_{L/2}^{x}\sigma_{L/2+1}^{x}$, a quartic structure can be seen as $\mathrm{tr}(AH^{m})\neq 0$ only for m≥4. This is fitted by $f_{A}^{\left(4\right)}\left(0\right)E_{n}^{4}/\left(4!L^{4}\right)$ (black dashed lines). With $\sigma_{l}^{y}$ observables, the expected structure is hexic (polynomial of sixth degree) because $\mathrm{tr}(AH^{m})\neq 0$ only for m≥6. Though it is less clear, we fit it with the expected scaling $f_{A}^{\left(6\right)}\left(0\right)E_{n}^{6}/\left(6!L^{6}\right)$ using the black dashed lines. For the $\sigma^{z}$ observable, we find a parabolic structure in accordance with our prediction as $\mathrm{tr}(AH^{2})\neq 0$, whereas tr(AH)=0. Since we consider systems of size L=14, the results in Figure 1j–l are partially affected by finite-size effects. Despite this, the numerics are aligned with our theoretical predictions.

To summarize this section, we observe clearly that the diagonal elements of operators can have a very different dependence as a function of energy near zero. In particular, we have numerically verified the prediction that $A_{nn}∼1/L^{p}$, where *p* is the lowest positive integer such that $\mathrm{tr}(AH^{p})\neq 0$.

### 4.3. Scaling of the Infinite Time Value of OTOC

**Figure 2:**
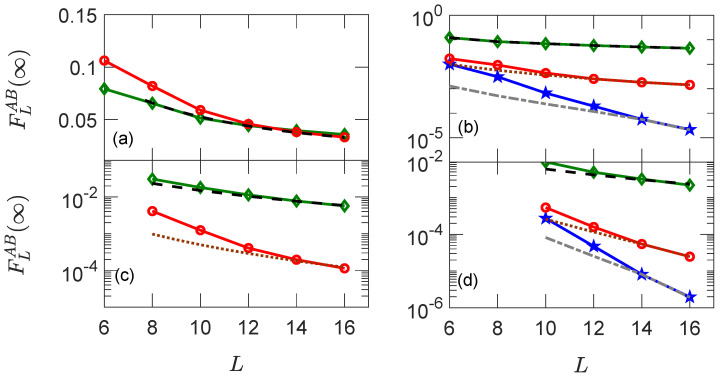
Infinite time values of OTOC corresponding to the single-site observables with $A=\sigma_{L/2-1}^{\alpha }$, $B=\sigma_{L/2}^{\alpha }$ panel (**a**), double-site observables with $A=\sigma_{L/2-2}^{\alpha }\sigma_{L/2-1}^{\alpha }$, $B=\sigma_{L/2}^{\alpha }\sigma_{L/2+1}^{\alpha }$ (**b**), triple-site observables with $A=\sigma_{L/2-3}^{\alpha }\sigma_{L/2-2}^{\alpha }\sigma_{L/2-1}^{\alpha }$, $B=\sigma_{L/2}^{\alpha }\sigma_{L/2+1}^{\alpha }\sigma_{L/2+2}^{\alpha }$ (**c**), and quadruple-site observables with $A=\sigma_{L/2-4}^{\alpha }\sigma_{L/2-3}^{\alpha }\sigma_{L/2-2}^{\alpha }\sigma_{L/2-1}^{\alpha }$, $B=\sigma_{L/2}^{\alpha }\sigma_{L/2+1}^{\alpha }\sigma_{L/2+2}^{\alpha }\sigma_{L/2+3}^{\alpha }$ (**d**). Green lines with diamonds are for observables involving only $\sigma_{l}^{z}$(α=z) operators, red lines with circles are for $\sigma_{l}^{x}$(α=x), and blue lines with stars are for $\sigma_{l}^{y}$(α=y) operators, respectively. Black-dashed, brown-dotted, and grey-dashed dotted lines are the fits for $\sigma_{l}^{z}$, $\sigma_{l}^{x}$, and $\sigma_{l}^{y}$ observables. Here, $J_{z}=1$, $h_{x}=0.9$, and $h_{z}=0.809$.

In Figure 2, we show numerical confirmation that, given the minimum positive integer $p_{c}$ such that $\mathrm{tr}(AH^{p_{c}})\neq 0$ or $\mathrm{tr}(BH^{p_{c}})\neq 0$, then $F_{L=\infty }^{AB}\left(t\right)\propto 1/L^{p_{c}}$. In each of the panels, we show how the infinite time value of the OTOC $F_{L}^{AB}\left(t=\infty \right)$ varies as a function of the system size *L*. In the different panels, we will focus on single-site, panel (a); two-site, panel (b), three-site, panel (c); and four-site, panel (d), observables. In each panel, the red line with circles corresponds to α=x, blue with stars to α=y, and green with diamonds to α=z. In panel Figure 2a, we plot the infinite time values of OTOC with single-site observables $A,B=\sigma_{l}^{\alpha }$, where l=L/2 for observable *B* and l=L/2−1 for observable *A*. We see that these observables have $p_{c}=1$, and hence they follow 1/L scaling, as shown by dashed line. $\sigma_{l}^{y}$ has no overlap with any local conserved quantities, and hence the diagonal elements as well as the infinite time values of OTOC are zero. Figure 2b is for double-site observables Equation (19), where l=L/2 for observable *B* and l=L/2−2 for observable *A*. We compare the numerical results with fitted lines, in particular with $1/L^{2}$ (dotted), $1/L^{3}$ (dashed dotted lines), and 1/L (dashed), respectively, corresponding to operators with $p_{c}=2,3$ and 1. We note that due to the small value of the overlap of $\sigma_{j}^{y}\sigma_{j+1}^{y}$ with the Hamiltonian, the expected scaling is followed only at larger system sizes. In panel (c), we plot the triple-site observables Equation (20), where j=L/2−3 for *A* and j=L/2 for *B*. Fitted lines are for $1/L^{2}$ and $1/L^{3}$ scalings, as expected, since α=2 and 3, respectively. Since the diagonal elements of $\sigma_{j}^{y}\sigma_{j+1}^{y}\sigma_{j+2}^{y}$ are zero, the infinite time value of the OTOC $F_{L}^{AB}\left(t=\infty \right)$ is zero. Panel (d) is for quadruple-site observables Equation (21), where j=L/2−4 for *A* and j=L/2 for *B*. The expected scalings are $p_{c}=4,6,2$. However, due to the fact that the observables have a large support at initial time, we see that the correct scaling of $1/L^{4},1/L^{6},1/L^{2}$ is followed only at large system sizes.

### 4.4. Dynamics of OTOCs

**Figure 3:**
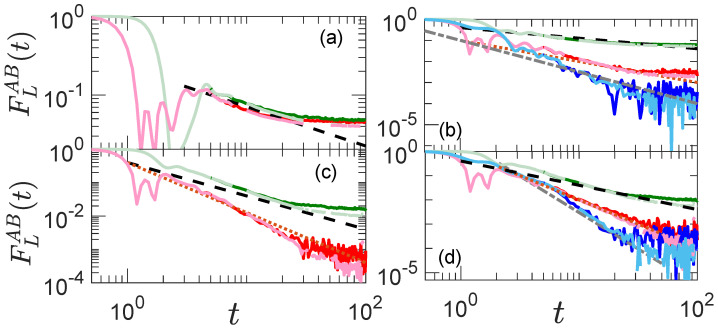
Time evolution of OTOC corresponding to the single-site observables with $A=\sigma_{L/2-1}^{\alpha }$, $B=\sigma_{L/2}^{\alpha }$ panel (**a**), double-site observables with $A=\sigma_{L/2-2}^{\alpha }\sigma_{L/2-1}^{\alpha }$, $B=\sigma_{L/2}^{\alpha }\sigma_{L/2+1}^{\alpha }$ (**b**), triple-site observables $A=\sigma_{L/2-3}^{\alpha }\sigma_{L/2-2}^{\alpha }\sigma_{L/2-1}^{\alpha }$, $B=\sigma_{L/2}^{\alpha }\sigma_{L/2+1}^{\alpha }\sigma_{L/2+2}^{\alpha }$ (**c**), and quadruple-site observables $A=\sigma_{L/2-4}^{\alpha }\sigma_{L/2-3}^{\alpha }\sigma_{L/2-2}^{\alpha }\sigma_{L/2-1}^{\alpha }$, $B=\sigma_{L/2}^{\alpha }\sigma_{L/2+1}^{\alpha }\sigma_{L/2+2}^{\alpha }\sigma_{L/2+3}^{\alpha }$ (**d**). Green lines are for observables involving only $\sigma_{l}^{z}$(α=z) operators, red lines are for $\sigma_{l}^{x}$(α=x), and blue lines are for $\sigma_{l}^{y}$(α=y) operators respectively. Black-dashed, brown-dotted, and grey-dashed dotted lines are the fits for $\sigma_{l}^{z}$, $\sigma_{l}^{x}$, and $\sigma_{l}^{y}$ observables discussed in the text. Here, L=14 for lighter shades and L=12 for darker shades and $J_{z}=1$, $h_{x}=0.9$, and $h_{z}=0.809$.

We study the dynamics of OTOC in Figure 3, where each panel reflects the same case analyzed in the corresponding panel of Figure 2. Green lines are for observables involving only $\sigma_{l}^{z}$ operators, red lines are for $\sigma_{l}^{x}$, and blue lines are for $\sigma_{l}^{y}$ operators, respectively. In these plots, we need to study the long-time evolution. We thus need to disregard initial transients. At the same time, though, our results are affected by finite size, so we would need to concentrate on long yet intermediate times to evaluate the relaxation of the OTOC over time. Light shades are for L=14, and dark shades for L=12. Black-dashed, brown-dotted, and grey-dashed dotted lines are the fits for $\sigma_{l}^{z}$, $\sigma_{l}^{x}$, and $\sigma_{l}^{y}$ observables. Figure 3a is for single-site observables, as in Equation (18). We have already seen that since tr(OH)≠0, for (O=A,B), then the infinite time value of OTOC $F_{L}^{AB}\left(t=\infty \right)$ scales as 1/L. From our discussion at the end of Section 3.2, we thus expect that $F^{AB}\left(t\right)\propto 1/t^{1/2}$, and the numerical result of the dynamics, is well fitted by the black dashed line proportional to $t^{1/2}$.

In Figure 3b, we study the two-site observables of Equation (19) with l=L/2 for observable *B* and l=L/2−2 for observable *A*. As already discussed, the lowest order terms that have non zero values in the Taylor expansion for these observables are 1,2 and 3, respectively, for the $\sigma_{l}^{z},\sigma_{l}^{x},\sigma_{l}^{y}$ observables. In Figure 2b, we showed the scaling of the infinite-time OTOC for these observables as 1/L, $1/L^{2}$, $1/L^{3}$. Here, we would thus expect a scaling with times of $1/t^{1/2}$, 1/t, and $1/t^{3/2}$, as shown in the plots by dotted, dashed dotted, and dashed lines, respectively. We study the evolution of three-site observables of Equation (20) in Figure 3c. Here, l=L/2−3 for *A* and l=L/2 for *B*. Fitted lines are for 1/t and $1/t^{3/2}$ scaling as expected since tr(OH)≠0 for $\sigma_{l}^{z}$ and $\mathrm{tr}(OH^{2})\neq 0$ for the $\sigma_{l}^{x}$ observable. Panel (d) shows the dynamics for four site observables with l=L/2−4 for *A* and l=L/2 for *B*. The expected scaling is 1/t, $1/t^{2}$, and $1/t^{3}$ for α=z,x and *y*, respectively, whose operators for the corresponding critical exponent $p_{c}$ that gives non-zero overlap are 2,4, and 6.

## 5. Conclusions

OTOCs have been studied as a probe for quantum information scrambling. Slow, algebraic scrambling has been reported in systems with local conserved quantities [34,38,39,40].

In this paper, we showed that the higher the exponent at which ones elevates the Hamiltonian in order to have a non-zero overlap with the operators in the OTOC, the faster is the relaxation of the OTOC over time. Furthermore, if there is an exponent such that the overlap is non-zero, then the relaxation, even if it appears to be fast, is bounded to be, at the fastest, algebraic, and only if there is no overlap with any power of the Hamiltonian (or other conserved quantities), then the relaxation can be exponential.

From our results, it follows that considering single-site operators in the OTOC, and a local Hamiltonian with only a single site and nearest neighbours term, relaxation can only take a limited set of exponents. It is thus necessary to consider operators with larger support, such as two-site, three-site, and four-site operators, to observe a larger variety and magnitude of relaxation exponents. This, however, leads to the difficulty of studying the relaxation numerically due to more pronounced finite-size effects when studying operators with larger support. Future developments in numerical methods could help to test our results for larger systems.

In order to derive these results, we also found a relation between the first non-zero derivative of the function representing the diagonals of an operator in the energy basis and the first non-zero exponent of the Hamiltonian (which has non-zero overlap with the operators of the OTOC). Future works could extend these results to time-dependent systems with other types of conserved quantities.

## Data Availability

The data that support the findings of this study are available from the corresponding author upon reasonable request.
